# Surface Quality Improvement by Using a Novel Driving System Design in Single-Side Planetary Abrasive Lapping

**DOI:** 10.3390/ma14071691

**Published:** 2021-03-30

**Authors:** Zhenzhen Chen, Donghui Wen, Jianfei Lu, Jie Yang, Huan Qi

**Affiliations:** 1 Key Laboratory of Special Purpose Equipment and Advanced Processing Technology, Ministry of Education and Zhejiang Province, Zhejiang University of Technology, Hangzhou 310023, China; czzjudy@zjut.edu.cn (Z.C.); jieyangzjut@163.com (J.Y.); 2 Zhejiang Chendiao Machinery Co. Ltd., Jinyun 321400, China; zjsd@zjsd.com

**Keywords:** abrasive lapping, irrational rotation speed ratio, surface quality, material removal uniformity

## Abstract

For the traditional single-side planetary abrasive lapping process particle trajectories passing over the target surface are found to be periodically superposed due to the rational rotation speed ratio of the lapping plate to workpiece that could affect the material removal uniformity and hence its surface quality. This paper reports on a novel driving system design with combination of the tapered roller and contact roller to realize the irrational rotation speed ratio of the lapping plate to workpiece in the single-side planetary abrasive lapping process for the improvement of surface quality. Both of the numerical and experimental investigations have been conducted to evaluate the abrasive lapping performance of the novel driving system. It has been found from the numerical simulation that particle trajectories would theoretically cover the whole target surface if the lapping time is long enough due to their non-periodic characteristics, which can guarantee the uniformity of material removal from the surface of workpiece with relatively high surface quality. The encouraging experimental results underline the potential of the novel driving system design in the application of the single-side planetary abrasive lapping for the improvement of the surface quality in terms of surface roughness and material removal uniformity.

## 1. Introduction

Abrasive machining technology as one of the non-traditional machining technologies has been extensively used for the precision lapping of planar surfaces [1,2] and polishing of curved surfaces [3,4], and to fabricate complicated parts on a variety of materials [5,6], especially the hard-to-machine materials, due to its extinct advantages of high flexibility and efficiency over other traditional machining technologies, such as cutting, drilling and milling, with geometric tools [7,8,9]. To be specific, abrasive polishing technology by using the mixture of abrasive particles and fluid is usually used to precision polishing of the complicated structures with free-form surfaces that cannot achieved by conventional methods. For instance, the 3D printing parts, which are formed by the melting and bonding powders under the high-energy laser [10], should be pre-polished by abrasive flow to obtain a better surface finish before they can be used in industry [11]. In addition, abrasive lapping technology by using the abrasive particles embedded on the lapping plate is often employed for the precision lapping of large and planar surfaces with high efficiency and quality, so that it would reduce the cost and time for the following ultra-precision polishing process, such as the chemical mechanical polishing process [12], to obtain the ultra-smooth planar surfaces with relatively low surface roughness and surface damage [13]. However, according to the Preston theory the lapping time, pressure and relative velocity between the lapping plate and workpiece would affect the material removal process; so how to control the uniformity of the material removal from the target surface during the abrasive lapping process becomes the major issue that also attracts much attention from researchers.

The planetary abrasive lapping process by means of the simultaneous rotation of the lapping plate and workpiece has been proposed to overcome the non-uniform distribution of their relative velocity and abrasive particle trajectories passing over the target surface at a given lapping pressure, so that kinematics involved in this process need to be addressed to understand the abrasive particle trajectories. Li et al. [14] proposed a generic analytical model of the periodicity in the single-side planetary abrasive lapping process to investigate the distribution of the abrasive particle trajectories moving on the target surface and to provide guidance for both lapping parameter optimization and lapping pad design for industrial purpose. Yuan et al. [15] investigated the kinematics and trajectory of abrasive particles involved in the both-sides planetary abrasive lapping process and proposed the optimized strategy for the improvement of the material removal uniformity on the surface of the bearing roller. In these studies, they only considered the rational rotation speed ratio of the lapping plate to workpiece and explored related kinematics to evaluate and optimize the distribution of the particle trajectories by properly controlling the processing parameters, while Wen et al. [16] first proposed the concept of the irrational rotation speed ratio when they employed the single-side planetary abrasive lapping technology to fabricate interdigitated micro-channels on bipolar plates and found that after a long lapping time the particle trajectories were not superposed periodically. However, the underlying science and its application for the improvement of the abrasive lapping performance have not been investigated yet.

In this paper, a novel driving system is first designed to realize the irrational rotation speed ratio of the lapping plate to workpiece in the single-side planetary abrasive lapping machine and its working mechanism is theoretically analyzed. Then, to improve the surface quality the distribution of particle trajectories moving on the surface of the workpiece by using the novel driving system is numerically studied to assess its material removal uniformity. Finally, a set of the single-side planetary abrasive lapping tests on single-crystal silicon wafers by employing the novel driving system and the traditional driving system, respectively, are carried out to experimentally evaluate its lapping performance.

## 2. Evaluation of Surface Quality with the Novel Driving System

### 2.1. Mechanism of the Novel Driving System to Realize the Irrational Rotation Speed Ratio

**Figure 1:**
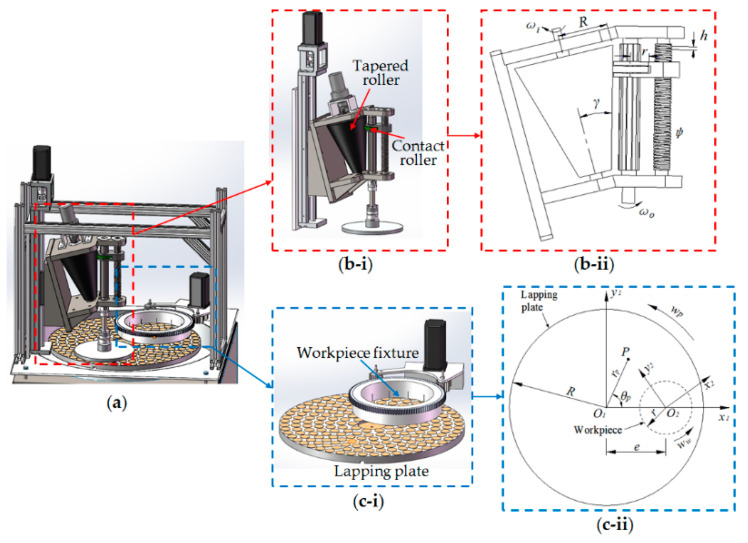
(**a**) Proposed single-side planetary lapping machine with irrational rotation speed ratio of lapping plate to workpiece; (**b-i**) the novel driving system design with a tapered roller and contact roller and (**b-ii**) its working mechanism; (**c-i**) the single-side eccentric abrasive lapping device and (**c-ii**) its working mechanism.

The design novelty of the proposed single-side planetary abrasive lapping machine, as shown in Figure 1, is to realize the irrational rotation speed ratio of the lapping plate to workpiece (*w_p_*/*w_w_*). This design consists of a novel driving system with a tapered roller and contact roller (Figure 1b-i) and a normal single-side planetary lapping device (Figure 1c-i), in which the working mechanism of the combination of the tapered roller and contact roller can be analyzed according to Figure 1b-ii as is detailed below.

The input rotation speed of the tapered roller (*w_i_*) was driven by a DC servo motor, and the press fitting friction between the tapered roller and contact roller would cause the rotation of the contact roller simultaneously, where the spline shaft was connected with the contact roller by the rectangle spline and it could rotate along the contact roller as well by the torque transmission. The fixture of the workpiece was connected to the bottom of the spline shaft, so that the output rotation speed of the spline shaft (*w_o_*) would be equal to the rotation speed of the workpiece (*w_w_*). Further, in Figure 1b-ii the contact roller was connected with the leading screw to realize its linear movement by adjusting the rotation angle of the screw rod, and thus, due to the changes of the relative position between the tapered roller and contact roller it could achieve the variations of the rotation speed ratio of the output to the input (*w_o_*/*w_i_*) in this novel driving system. To be specific, the rotation speed ratio, *i_oi_* = *w_o_*/*w_i_*, can be analyzed and calculated from Figure 1b-ii:

(1) $$i_{oi}=\frac{\omega_{o}}{\omega_{i}}=\frac{2\pi R-\phi h\sin\gamma }{2\pi r}$$

where *R* is the radius of the tapered roller, *r* is the radius of the contact roller, *h* is the screw travelling distance, *φ* is the rotation angle of the screw rod and *γ* is the half conical angle of the tapered roller. Since the rotation speed of the spline shaft (*w_o_*) is equal to the rotation speed of the workpiece (*w_w_*), the rotation speed ratio (*i_pw_*) of the lapping plate to workpiece (*w_p_*/*w_w_*) can be expressed as:

(2) $$i_{pw}=\frac{\omega_{p}}{\omega_{w}}=\frac{\omega_{p}}{i_{oi}\omega_{i}}=\frac{\omega_{p}}{\omega_{i}}\frac{2\pi r}{2\pi R-\phi h\sin\gamma }$$

In this novel driving system, some parameters of designed structures have been determined which include *R* = 123 mm, *r* = 45 mm, *h* = 2.5 mm and *γ* = 15°. If the input rotation speed (*w_i_*) driven by the DC servo motor assumed to be equal to the rotation speed of the lapping plate (*w_p_*) driven by the lapping machine, according to Equation (2) the variations of the *i_pw_* can be calculated by adjusting the rotation angle of the screw rod, *φ* and the results is shown in Table 1. It can be seen from Table 1 that the irrational rotation speed ratio (*i_pw_*) of the lapping plate to workpiece (*w_p_*/*w_w_*), such as 0.3819… and 0.4634…, can be realized by employing the novel driving system as designed in Figure 1.

### 2.2. Uniformity Assessment of Particle Trajectories Moving on the Surface of Workpiece by Using the Novel Driving System

Figure 1c-ii shows the working mechanism of the single-side planetary abrasive lapping process with schematic representation of its kinematics, and according to previous study [16] the trajectory of any point *P*(*r_p_*, *θ_p_*) fixed on the lapping plate when it moves along the surface of workpiece can be found from:

(3) $$x=r_{p}\cos\left(\theta_{p}+\omega_{p}t-\omega_{w}t\right)-e\cos\left(\omega_{w}t\right)$$

(4) $$y=r_{p}\sin\left(\theta_{p}+\omega_{p}t-\omega_{w}t\right)+e\sin\left(\omega_{w}t\right)$$

It can be found from Equation (3) that particle trajectories on the workpiece are related to the original position of the abrasive particle fixed on the lapping plate, the eccentric distance (*e*) between *O_1_* and *O_2_*, the rotation speed of the lapping plate (*w_p_*) and workpiece (*w_w_*). The numerical method is a powerful tool that is usually employed to address complicated problems that cannot be solved directly by an experiment [17,18], hence, by the assistance of Matlab the trajectories of single abrasive particle and multiple abrasive particles moving on the target surface can be simulated under different values of *i_pw_*, including the rational and irrational numbers, respectively. Thus, considering the experimental work to be given later in this paper the processing parameters involved in the simulation were set as follows: *R_p_* = 230 mm, *r_w_* = 90 mm, *e* = 130 mm and *w_p_* = 10 RPM, as shown in Figure 1c-ii.

**Figure 2:**
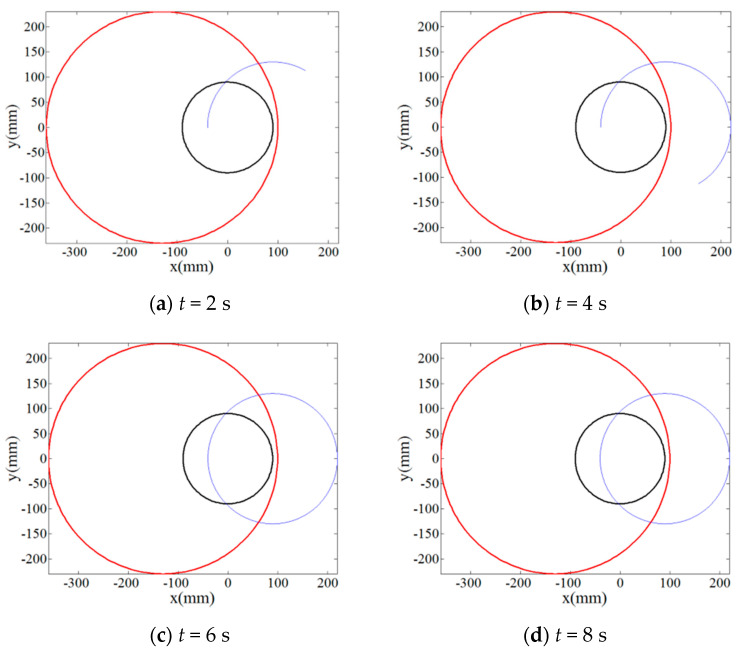
Relationship between trajectory of the single abrasive particle on target surface at *i_pw_* = 1 and lapping time (**a**) *t* = 2 s; (**b**) *t* = 4 s; (**c**) *t* =6 s; (**d**) *t* = 8 s (red lines represent the profile of the lapping plate, black lines represent the profile of the workpiece, and blue lines represent the trajectory of the single abrasive particle on the target surface).

**Figure 3:**
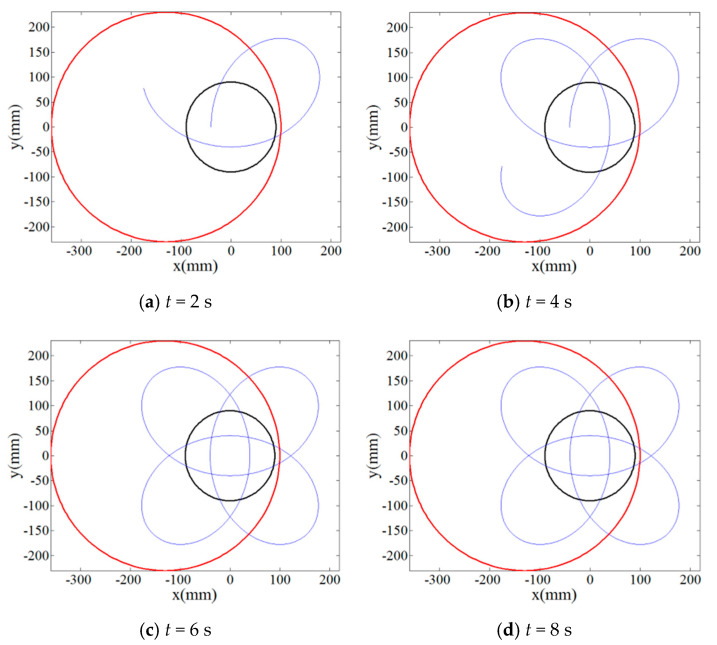
Relationship between trajectory of the single abrasive particle on target surface at *i_pw_* = 4/3 and lapping time (**a**) *t* = 2 s; (**b**) *t* = 4 s; (**c**) *t* =6 s; (**d**) *t* = 8 s.

**Figure 4:**
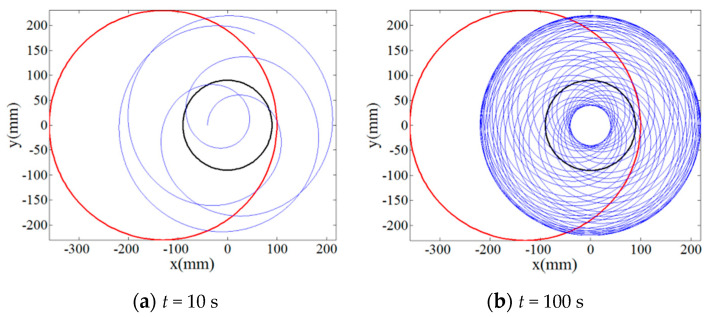

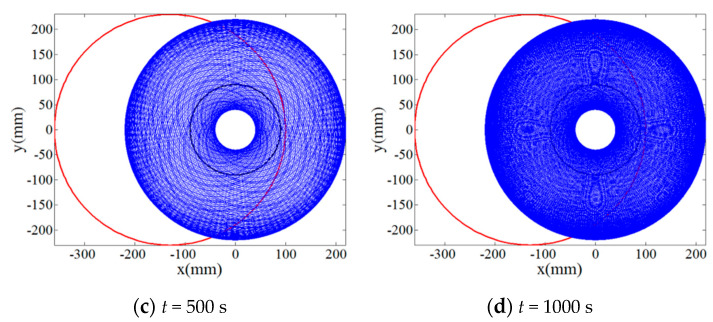
Relationship between trajectory of the single abrasive particle on target surface at *i_pw_* = 0.3819… and lapping time (**a**) *t* = 10 s; (**b**) *t* = 100 s; (**c**) *t* = 500 s; (**d**) *t* = 1000 s.

**Figure 5:**
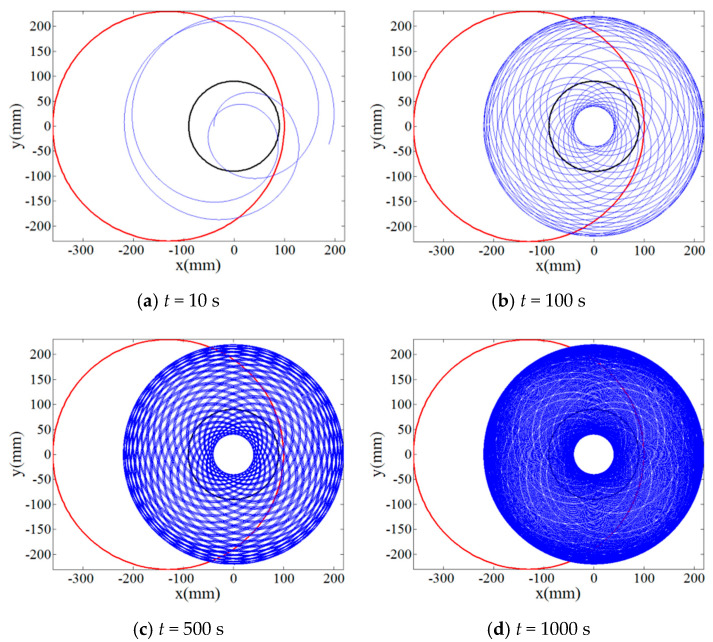
Relationship between trajectory of the single abrasive particle on target surface at *i_pw_* = 0.4634… and lapping time (**a**) *t* = 10 s; (**b**) *t* = 100 s; (**c**) *t* = 500 s; (**d**) *t* = 1000 s.

The single abrasive particle located at the polar position of *r_p_* = 90 mm, *θ_p_* = 0° on the lapping plate was first employed to numerically investigate the effect of rotation speed ratios of the lapping plate to workpiece, *i_pw_* = 1, 4/3, 0.3819… and 0.4634…, on its trajectory moving along the target surface according to the Equation (3). Figure 2 and Figure 3 show the result on the relationship between the trajectory of the single abrasive particle on target surface and lapping time under the rational numbers of *i_pw_*, i.e., 1 and 4/3, and it can be indicated from these figures that the periodic and closed-loop trajectory is formed if the lapping time is long enough. By contrast, even after a long lapping time of 1000 s the trajectory of single abrasive particle moving on the target surface under the irrational numbers of *i_pw_*, i.e., 0.3819… and 0.4634…, is not periodic and superposed as can be depicted from Figure 4 and Figure 5. Thus, the non-periodic trajectory of single abrasive particle moving on the surface of workpiece is expected to improve the material removal uniformity and surface quality.

**Figure 6:**
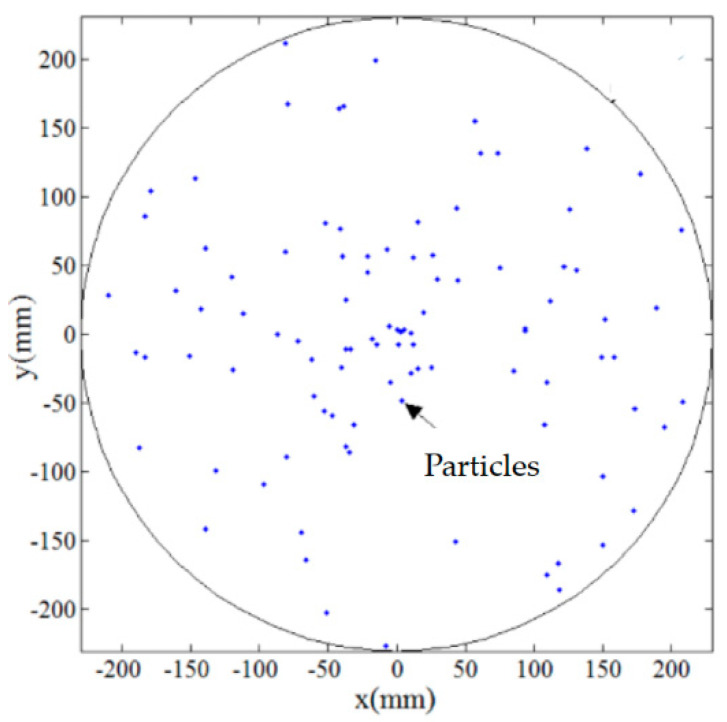
Random distribution of 100 abrasive particles on the lapping plate.

**Figure 7:**
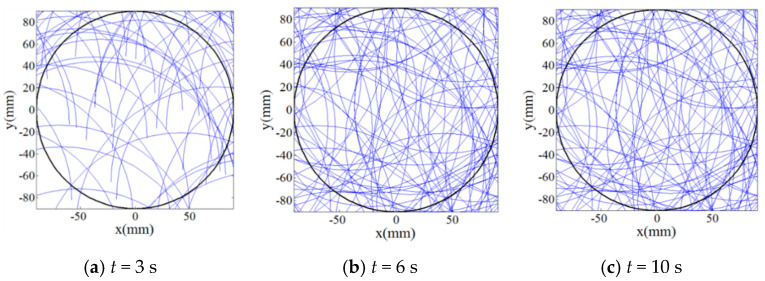
Relation between trajectories of multiple abrasive particles on target surface at *i_pw_* = 1 and lapping time (**a**) *t* = 3 s; (**b**) *t* = 6 s; (**c**) *t* = 10 s (black lines represent the profile of the workpiece and blue lines represent trajectories of multiple abrasive particles on the target surface).

**Figure 8:**
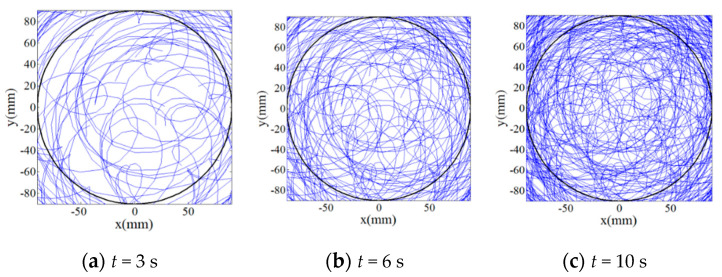
Relationship between trajectories of multiple abrasive particles on target surface at *i_pw_* = 0.3819… and lapping time (**a**) *t* = 3 s; (**b**) *t* = 6 s; (**c**) *t* = 10 s.

Then, in order to qualitatively and quantitatively evaluate the uniformity of trajectories by the multiple abrasive particles, 100 abrasive particles are randomly distributed on the lapping plate as presented in Figure 6 and their trajectories moving on the surface of the workpiece have been analyzed under rational and irrational numbers of *i_pw_*, i.e., 1 and 0.3819…, respectively. It can be seen from Figure 7 that when the lapping time is more than 6 s a minor variation of the trajectories can be found as compared with the lapping time of 10 s, and it demonstrates that when the lapping time is long enough the trajectories of multiple abrasive particles driven by the rational rotation speed ratio, *i_pw_* = 1, will be periodically superposed. However, it is interesting to notice from Figure 8 that the density of trajectories on target surface is increasingly large with an increment of lapping time at irrational rotation ratio, *i_pw_* = 0.3819…, as such, by further increasing the lapping time the trajectories of multiple abrasive particles are expected to cover the whole surface of the workpiece due to their non-periodic characteristics. Therefore, it is found from the qualitative study that within the same lapping time the distribution of particle trajectories on the target surface driven by the irrational number of *i_pw_* is denser and uniform than that driven by the rational number of *i_pw_* under the same processing parameters.

**Figure 9:**
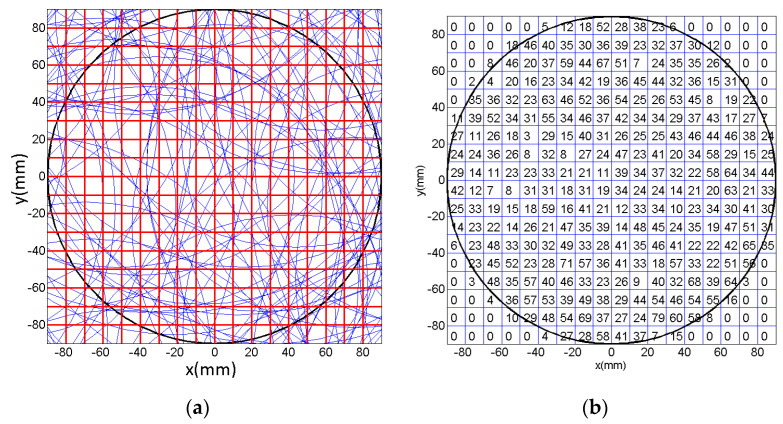
(**a**) Schematic representation of the divided squares on the surface of workpiece and (**b**) the total number of trajectories passing over each square at at *i_pw_* = 1 and lapping time *t* = 6 s.

Further, a quantitative analysis [19] has been also conducted to assess the uniformity of trajectories by multiple abrasive particles on workpiece by the assistance of Matlab according to the following steps:

(1) To divide the target surface into small squares with density of 10 mm × 10 mm, as shown in Figure 9a, the red line squares;

(2) To count the numbers of trajectories passing over each square with *Q_i_* (*i* = 1, 2,…, *N*), as shown in Figure 9b, the total number of trajectories passing over each square at at *i_pw_* = 1 and lapping time *t* = 6 s.

(3) To calculate the Standard Deviation (*S_Q_*) of all the values of *Q_i_* according to the following equation:

(5) $$S_{Q}=\sqrt{\frac{\sum_{i=1}^{n}{\left(Q_{i}-\bar{Q}\right)}^{2}}{n-1}}$$

where $\bar{Q}$ is the average value of *Q_i_*;

(4) To calculate the Coefficient of Variation (*CV*) of *Q_i_* according to the following equation:

(6) $$CV=\frac{S_{Q}}{\bar{Q}}$$

**Figure 10:**
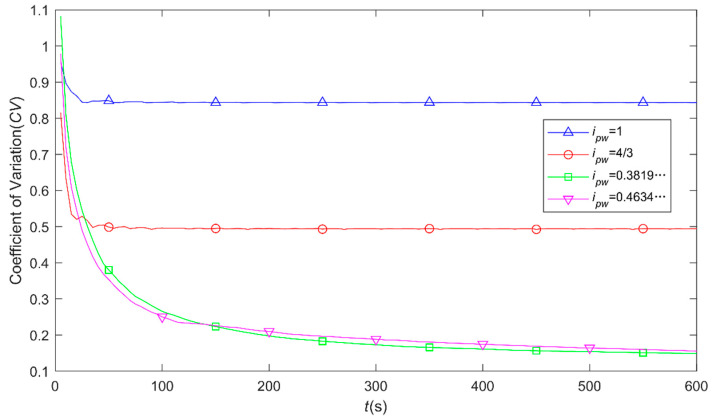
Variations of *CV* values by increasing the lapping time under different rotation speed ratios.

To consider the total lapping time of 600 s and the sampling step of 0.001 s, the results on the relation between the *CV* values and lapping time with respect to the different rotation speed ratios, *i_pw_*, of 1, 4/3, 0.3819… and 0.4634… are shown in Figure 10. As can be seen from Figure 10 that when the rotation speed ratios of the lapping plate to workpiece are rational numbers the values of *CV* rapidly decrease to be constant within the lapping time of about 100 s, and there is a large *CV* value difference between the *i_pw_* of 1 and 4/3. It is indicated that with rational number of *i_pw_* particle trajectories moving on the target surface would be periodically superposed after a certain lapping time and the relatively high values of *CV* means that the particle trajectories only pass over part of the target surface which could affect its material removal uniformity and hence, the surface quality. By contrast, the values of *CV* seem to gradually decrease until very small values with respect to the lapping time under the irrational rotation speed ratios of the lapping plate to workpiece, and there is no significant difference between the *i_pw_* of 0.3819… and 0.4634… in terms of *CV* values. As such, particle trajectories would theoretically cover the whole target surface if the lapping time is long enough, and it can guarantee the uniformity of material removal from surface of workpiece with relatively high surface quality as well.

The working mechanism of the novel driving system by combining a tapered roller and contact roller has been theoretically analyzed to realize the irrational rotation speed ratio of the lapping plate to workpiece, and uniformity assessment of particle trajectories moving on the target surface driven by this novel system has also been qualitatively and quantitatively investigated, in which the uniformity of material removal from the target surface is expected to be improved so that it would, in turn, improve its surface quality.

## 3. Performance Testing of the Novel Driving System

**Figure 11:**
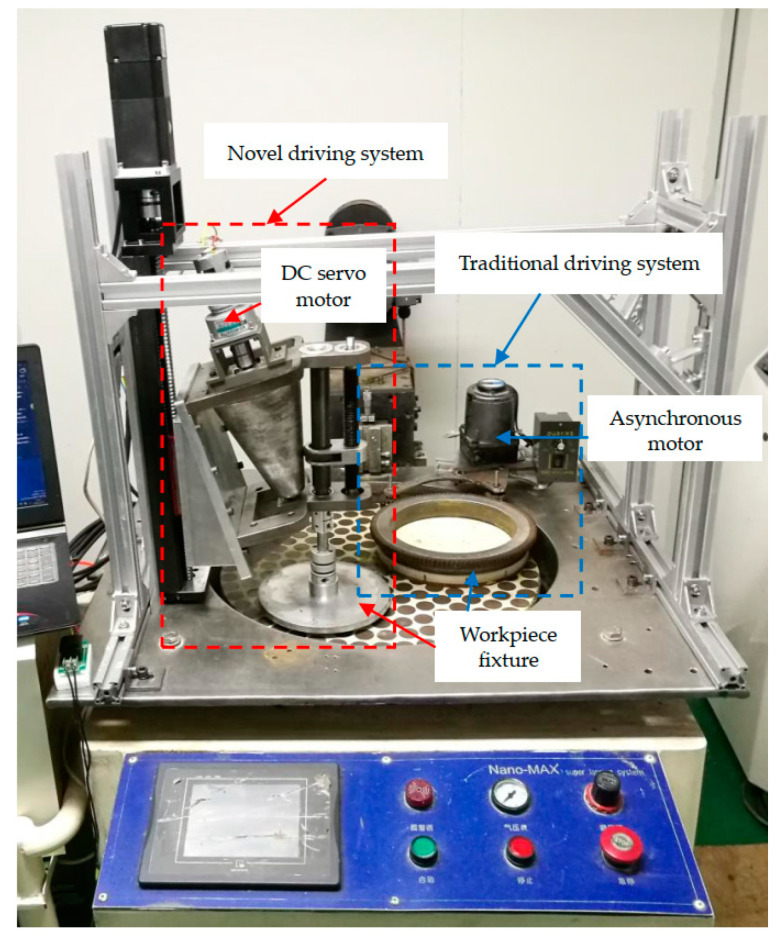
Experimental setup of the single-side planetary abrasive lapping test.

A set of the single-side planetary abrasive lapping tests on single-crystal silicon wafers by employing the novel driving system and the traditional driving system, respectively, were carried out to experimentally evaluate its machining performance. An in-house developed experimental device, as shown in Figure 11, was designed on the Nano-Max 9B single-side planetary abrasive lapping machine, in which the novel driving system was connected with a DC servo motor to realize the irrational rotation speed ratio of the lapping plate to workpiece, and the traditional driving system was connected with an asynchronous motor to realize the rational number of *i_pw_*.

**Figure 12:**
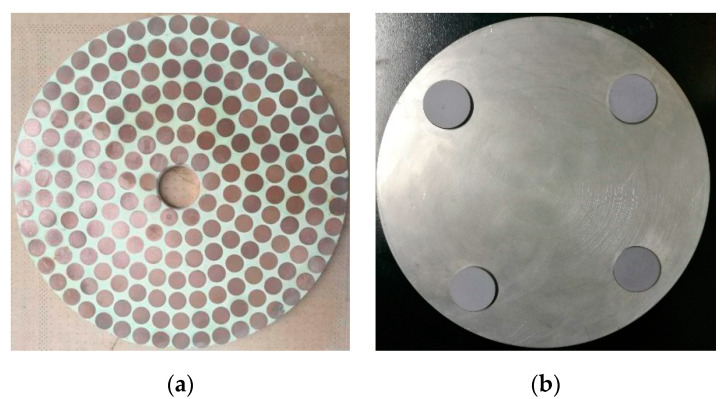
(**a**) The resin bond diamond lapping plate, and (**b**) the distribution of workpieces on the fixture.

The lapping plate (Maofeng Abrasive Material Co.Ltd, Dongguan, China) was made of resin bond (phenolic resin powder mixed with in a certain proportion of chromium oxide, zinc oxid, raphite and silicon carbide) diamond with mesh size of 1000^#^ and concentration of 75%, and its dimensions were 460 mm in outer diameter, 60 mm in inner diameter and 15 mm in thickness, as shown in Figure 12a. The flatness of the lapping plate is between 0.001 and 0.004 mm, and the roughness is between 0.1 and 1.2 μm, which is suitable for precision lapping of silicon wafer, structural ceramics and other brittle-hard materials. In order to eliminate the influence of random free abrasive and grinding pressure on the evaluation of material removal uniformity, such as warpage deformation, the single-crystal silicon wafer was selected as the workpiece with diameter of 25.4 mm and thickness of 2 mm, and in order to evaluate the material removal uniformity 4, 6, 8 and 10 pieces of workpiece were considered and distributed in equal angle with each other on the workpiece fixture with diameter of 180 mm as depicted in Figure 12b. Further, both of the rational and irrational numbers of *i_pw_*, i.e., 1, 4/3, 0.3819… and 0.4634…, were considered in experiment, and the lapping pressure on the workpiece and the rotation speed of lapping plate for each experimental condition were set as constants of 1.5 kg and 10 RPM, respectively.

The overall surface morphology was inspected by using the Keyence VHX-1000 3D Digital Microscope (Keyence Corp., Itasca, IL, USA), while the detail of surface characteristics, including surface roughness and surface profile accuracy, were obtained with the assistance of the Veeco Wyko NT9800 Optical Surface Profiler (Veeco, Tucson, AZ, USA). The material removal mass for each workpiece before and after the experiment was measured by the Shimadzu AUW220D micro-balance (Shimadzu, Kyoto, Japan) with resolution of 0.01 mg, and each test was repeated at least three times and the average data were used for the further analysis.

## 4. Results and Discussion

**Figure 13:**
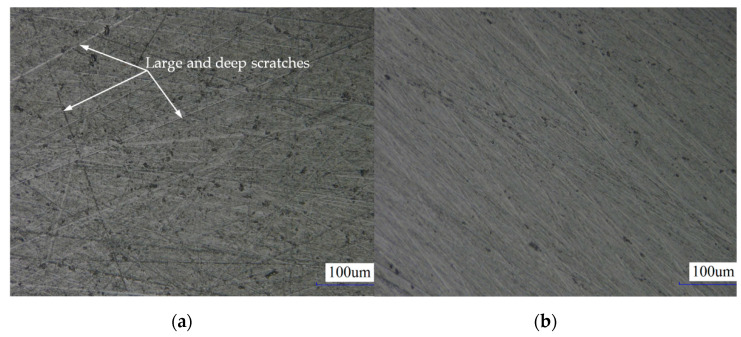
(**a**) Overall surface morphology at *i_pw_* = 1, and (**b**) overall surface morphology at *i_pw_* = 0.3819…, respectively.

Figure 13 shows the typical surface morphologies that are obtained from the single-side planetary abrasive lapping process at *i_pw_* = 1 and *i_pw_* = 0.3819…, respectively, under the lapping time of 1 h. It can be seen from Figure 13a that relatively large and deep scratches are found on the surface of workpiece, and they are formed due to the periodically superposed cutting actions by the abrasive particles. While by using the novel driving system to realize the irrational rotation speed ratio of the lapping plate to workpiece, *i_pw_* = 0.3819…, the surface seems to be smooth without significantly large scratches as illustrated in Figure 13b.

**Figure 14:**
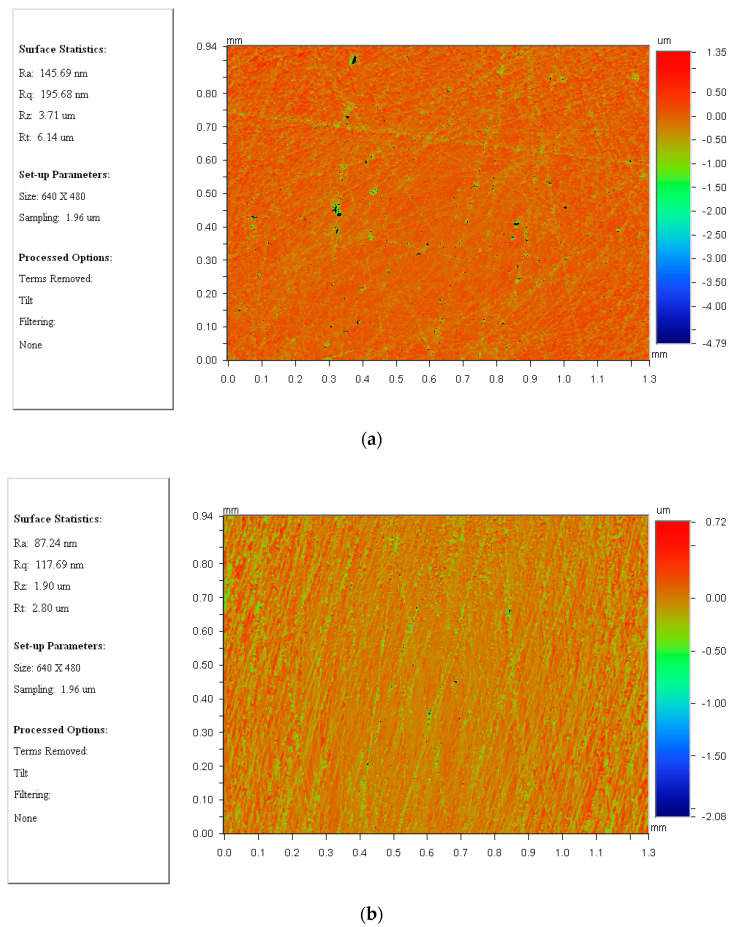
(**a**) 2D view of surface characteristics at *i_pw_* = 1, and (**b**) 2D view of surface characteristics at *i_pw_* = 0.3819…, respectively.

Further, surface characteristics, including surface roughness (*R_a_*), root-mean-square value of surface profile height (*R_q_*), maximum surface profile height (*R_z_*) and maximum peak-valley difference (*R_t_*), are observed with details as depicted in Figure 14. Obvious and deep scratches can be found from Figure 14a and only a part of the target surface has been lapped by the abrasive particles. However, clear lapped impressions which could almost cover the whole measured surface can be found from Figure 14b and they are evenly distributed in the target surface. Then, a quantitative analysis on the comparison between surface characteristics lapped at *i_pw_* = 1 and *i_pw_* = 0.3819… has been also conducted as shown in Table 2. It is found that, in general, the values of *R_a_*, *R_q_*, *R_z_* and *R_t_* measured from the target surface lapped at *i_pw_* = 0.3819… are less than that from *i_pw_* = 1, and to be specific the surface roughness, *R_a_*, has been found to be reduced to 87.24 nm which demonstrates that a better surface finish can be achieved by using the novel driving system. It is also interesting to note that the root-mean-square value of surface profile height, *R_q_*, is down to 117.69 nm at *i_pw_* = 0.3819… from 195.68 nm at *i_pw_* = 1, which indicates that the uniformity of the material removal from the target surface has been improved as well.

**Figure 15:**
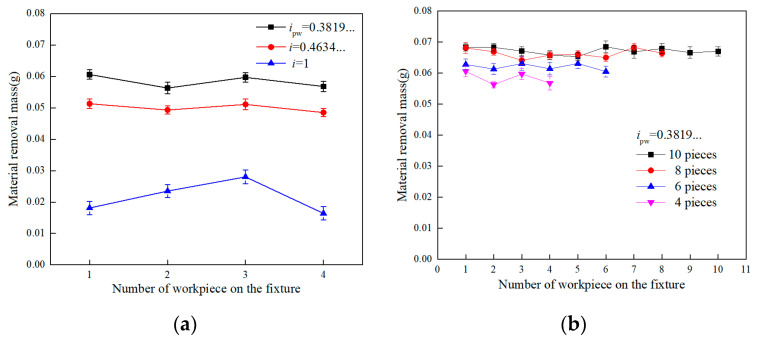
Effect of rotation speed ratios on: (**a**) material removal mass for each piece by considering 4 pieces of workpiece, and (**b**) material removal mass for each piece by considering 4, 6, 8 and 10 pieces of workpiece, respectively.

In addition, the analysis on the material removal mass of workpieces (lapping time of 1 h) distributed at different positions of the workpiece fixture has been carried out to experimentally evaluate the distribution of abrasive particles passing over the target surface and hence the material removal uniformity. It can be seen from Figure 15a that with irrational numbers of *i_pw_*, i.e., 0.3819… and 0.4634…, the material removal mass at each position of workpiece seems to be similar under the same experimental conditions while with a rational number of *i_pw_* = 1, the material removal mass at different positions of the workpiece is different. This finding is in agreement with the previous simulation result and verifies that the uniformity of particle trajectories moving on the surface of workpiece has been improved by using the irrational number of *i_pw_*. As such, the material removal uniformity from the target surface would be improved as well. Similar results can be found from Figure 15b, that by increasing the number of workpieces distributed on the workpiece fixture, as shown in Figure 12b, the material removal mass for each position of workpiece is still stable with irrational number of *i_pw_* = 0.3819…. Therefore, by qualitatively and quantitatively analyzing the surface morphology and characteristics it is found that the surface quality, including surface roughness and material removal uniformity, has been significantly improved by using the irrational number of *i_pw_* in the single-side planetary lapping process, and it could reduce the cost and time for the further ultra-precision polishing process with advanced robotics technology [20,21,22,23,24,25].

## 5. Conclusions

In this paper, a new concept of a driving system design with combination of the tapered roller and contact roller to realize the irrational rotation speed ratio of the lapping plate to workpiece, *i_pw_*, in the single-side planetary abrasive lapping process for the improvement of surface quality is presented. It has been found that the press fitting friction between the tapered roller and contact roller would cause the rotation of the contact roller simultaneously, where the spline shaft was connected with the contact roller by the rectangle spline and it could rotate along the contact roller as well by the torque transmission, and in this way by adjusting the rotation angle of the screw rod it could achieve the variations of the irrational rotation speed ratios of the output to the input in this novel driving system.

For concept validation, the uniformity assessment of particle trajectories moving on the target surface driven by this novel system has been qualitatively and quantitatively investigated. It is found from qualitative analysis that within the same lapping time the distribution of particle trajectories on the target surface driven by the irrational number of *i_pw_* is more dense and uniform than that driven by the rational number of *i_pw_* under the same processing parameters, and from the quantitative analysis that particle trajectories would theoretically cover the whole target surface if the lapping time is long enough, which can guarantee the uniformity of material removal from the surface of the workpiece with relatively high surface quality. Finally, an experimental study has been carried out to evaluate the abrasive lapping performance of the novel driving system. It has been found that not only does the surface roughness reduce to 87.24 nm, which demonstrates that a better surface finish can be achieved by using the novel driving system, but also that the material removal uniformity has been significantly improved by using the irrational number of *i_pw_* in the single-side planetary lapping process. Therefore, the encouraging results underline the potential for a novel driving system design in the application of the single-side planetary abrasive lapping for the improvement of the surface quality in terms of surface roughness and material removal uniformity.

## Figures and Tables

**Table 1:** Variations of the *i_pw_* by adjusting the rotation angle of the screw rod, *φ*.

*φ* (rad)	*i_pw_*	Approximate Value of *i_pw_*
16π	$\frac{45}{123-5\times \left(\sqrt{6}-\sqrt{2}\right)}$	0.3819…
80π	$\frac{45}{123-25\times \left(\sqrt{6}-\sqrt{2}\right)}$	0.4634…

**Table 2:** Comparison between surface characteristics at *i_pw_* = 1 and *i_pw_* = 0.3819….

*i_pw_*	*R_a_* (nm)	*R_q_* (nm)	*R_z_* (µm)	*R_t_* (µm)
1	145.69	195.68	3.71	6.14
0.3819…	87.24	117.69	1.9	2.8

## Data Availability

Data sharing not applicable.
